# Does all single infarction have lower risk of stroke recurrence than multiple infarctions in minor stroke?

**DOI:** 10.1186/s12883-018-1215-0

**Published:** 2019-01-08

**Authors:** Guangyao Wang, Jing Jing, Yuesong Pan, Xia Meng, Xingquan Zhao, Liping Liu, Hao Li, David Wang, Yongjun Wang, Yilong Wang, On behalf of the CHANCE Investigatores, Yongjun Wang, S. Claiborne Johnston, Yilong Wang, Xingquan Zhao, Zhimin Wang, Haiqin Xia, Guiru Zhang, Xudong Ren, Chunling Ji, Guohua Zhang, Jianhua Li, Bohua Lu, Liping Wang, Shutao Feng, Dali Wang, Weiguo Tang, Juntao Li, Hongtian Zhang, Guanglai Li, Baojun Wang, Yuhua Chen, Ying Lian, Bin Liu, Junfang Teng, Rubo Sui, Lejun Li, Zhiling Yuan, Dawei Zang, Zuneng Lu, Li Sun, Dong Wang, Liying Hou, Dongcai Yuan, Yongliang Cao, Hui Li, Xiuge Tan, Huicong Wang, Haisong Du, Mingyi Liu, Suping Wang, Qiuwu Liu, Zhong Zhang, Qifu Cui, Runqing Wang, Jialin Zhao, Jiewen Zhang, Jianping Zhao, Qi Bi, Xiyou Qi, Junyan Liu, Changxin Li, Ling Li, Xiaoping Pan, Junling Zhang, Derang Jiao, Zhao Han, Dawei Qian, Jin Xiao, Yan Xing, Huishan Du, Guang Huang, Yongqiang Cui, Yan Li, Lianyuan Feng, Lianbo Gao, Bo Xiao, Yibin Cao, Yiping Wu, Jinfeng Liu, Zhiming Zhang, Zhengxie Dong, Limin Wang, Li He, Xinchen Wang, Xueying Guo, Ming Wang, Xiaosha Wang, Jiandong Jiang, Renliang Zhao, Shengnian Zhou, Hao Hu, Maolin He, Fengchun Yu, Quping Ouyang, Jingbo Zhang, Anding Xu, Xiaokun Qi, Lei Wang, Fuming Shi, Fuqiang Guo, Jianfeng Wang, Fengli Zhao, Ronghua Dou, Dongning Wei, Qingwei Meng, Yilu Xia, Shimin Wang, Zhangcang Xue, Yuming Xu, Liping Ma, Chun Wang, Jiang Wu, Yifeng Du, Yinzhou Wang, Lijun Xiao, Fucong Song, Wenli Hu, Zhigang Chen, Qingrui Liu, Jiemin Zhang, Mei Chen, Xiaodong Yuan, Zhihui Liu, Guozhong Li, Xiaohong Li, Tingchen Tian

**Affiliations:** 1 Department of Neurology, Beijing Tiantan Hospital, Capital Medical University, Beijing, China; 2 China National Clinical Research Center for Neurological Diseases, Beijing, China; 3 Center of Stroke, Beijing Institute for Brain Disorders, Beijing, China; 4 Beijing Key Laboratory of Translational Medicine for Cerebrovascular Disease, Beijing, China; 5 Illinois Neurological Institute Stroke Network, Sisters of the Third Order of St. Francis Healthcare System, University of Illinois College of Medicine, Peoria, USA

**Keywords:** Minor stroke, Infarction patterns, Prognosis

## Abstract

**Background:**

Single acute infarction (SAI) usually had lower risk of stroke recurrence than multiple acute infarctions (MAIs) in minor stroke. To evaluate whether all SAI had lower risk of stroke recurrence than MAIs in minor stroke.

**Methods:**

We derived data from the imaging subgroup of the Clopidogrel in High-risk Patients with Acute Nondisabling Cerebrovascular Events (CHANCE) trial. Minor stroke were categorized into SAI and MAIs by infarction numbers in diffusion weighted imaging. SAI were classified as lacunar infarction and non-lacunar infarction. The outcome was stroke recurrence within one-year follow-up. We assessed the associations between infarction patterns and stroke recurrence using multivariable Cox regression models.

**Results:**

Overall, 834 patients with minor stroke were included in this subgroup, 553 SAI (381 lacunar infarction, 172 non-lacunar infarction) and 281 MAIs. The rate of stroke recurrence was 7.6%, 15.1% and 15.3% in lacunar infarction of SAI, non-lacunar infarction of SAI and MAIs at one year, respectively. Compared with MAIs, lacunar infarction of SAI had lower risk of stroke recurrence (hazard ratio [HR] 0.41, 95% confidence interval [CI] 0.21–0.80, *P* = 0.009), but not in non-lacunar infarction of SAI (HR 1.01, 95% CI 0.60–1.69, *P* = 0.98).

**Conclusions:**

Lacunar infarction of SAI have lower risk of stroke recurrence than MAIs, while non-lacunar infarction of SAI might have similar risk as MAIs. Except for the number of infarctions, size and location should also be considered to stratify risk of stroke recurrence in minor stroke.

**Trial registration:**

http://www.clinicaltrials.gov Unique identifier: NCT00979589. Date of registration: September 2009.

**Electronic supplementary material:**

The online version of this article (10.1186/s12883-018-1215-0) contains supplementary material, which is available to authorized users.

## Background

Minor stroke are the most common manifestations of acute cerebrovascular disease and the proportion of minor stroke in all ischemic stroke is approximately 50% [1]. Patients with minor stroke had higher risk of recurrence after symptom onset, especially in the early stage [2]. Recent studies suggested that vascular and neuroimaging parameters may improve risk stratification in minor stroke [3, 4]. TIA registry.org project showed that infarction patterns helped to stratify the risk of stroke recurrence within one year after minor stroke and patients with multiple acute infarctions (MAIs) had much higher risk of stroke recurrence than that with single acute infarction (SAI) or no acute infarction (NAI), indicating that MAIs was an important imaging marker to predict stroke recurrence [5]. However, several studies showed that there were different patterns in SAI and MAIs respectively corresponding to different stroke etiologies [6, 7] or mechanisms [8, 9]. Different stroke etiologies or mechanisms might lead to different risk of stroke recurrence [10–15]. Traditionally, SAI were classified according to the size and location of the infarction, while MAIs were classified according to the blood supply of different brain areas [6, 7]. However, it was unclear whether different infarction patterns of SAI and MAIs respectively had different risk of stroke recurrence after minor stroke and whether all SAI had lower risk of stroke recurrence than MAIs in minor stroke.

In the current study, deriving data from the imaging subgroup of the Clopidogrel in High-risk Patients with Acute Nondisabling Cerebrovascular Events (CHANCE) trial, we investigated whether among patients with SAI or MAIs whether different infarction patterns were associated with different risk of stroke recurrence. We further compared the risk of stroke recurrence in SAI with different infarction patterns to that of MAIs.

## Methods

### Overview of the CHANCE trial

The detailed design and methods of the CHANCE trial have been previously described [16, 17]. Briefly, CHANCE was a randomized, double-blind, placebo-controlled clinical trial conducted in 114 centers in China between October 2009 and July 2012. Totally, 5170 patients within 24 h of non-cardioembolic minor ischemic stroke or high-risk TIA onset were randomly assigned to either clopidogrel plus aspirin (clopidogrel at an initial dose of 300 mg, followed by 75 mg per day for 90 days, plus aspirin at 75 mg per day for the first 21 days) or placebo plus aspirin (75 mg per day for 90 days) group. The trial was approved by the Ethics Committee of Beijing Tiantan Hospital and all the participating hospitals. Written informed consent was obtained from all participants or their legal proxies. This study was registered at Clinical Trials.gov (registration number NCT00979589).

### Overview of the imaging substudy of the CHANCE trial

This imaging study was a prespecified substudy of the CHANCE trial. Briefly, 45 (39%) of 114 centers of the CHANCE trial were prospective recruited in the imaging substudy voluntarily. All patients were asked to complete the magnetic resonance (MR) examinations (3.0 or 1.5 Tesla) during hospitalization in this substudy. Patients with the following MR sequences were included in the substudy: T1-weighted imaging, T2-weighted imaging, diffusion-weighted imaging (DWI), and 3-dimensional (3D) time-of-flight magnetic resonance angiography (MRA). Those without baseline MR examination or any of the above sequences were excluded. The details of the CHANCE imaging substudy have been previously described [4, 18].

### Patient screening and image analysis

**Fig. 1:**
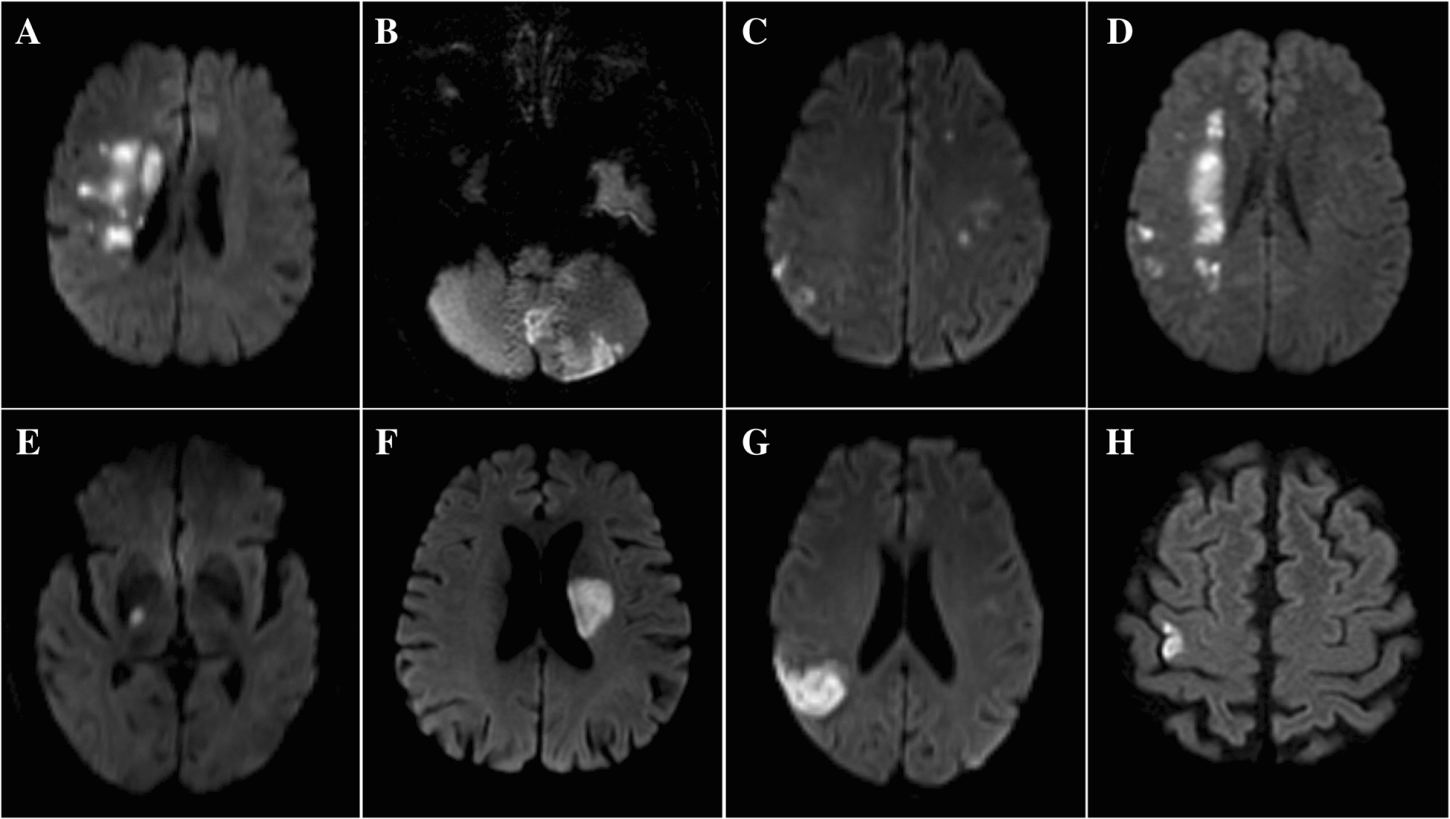
Infarction patterns of single acute infarction and multiple acute infarctions. Multiple acute infarctions. **a** Unilateral anterior circulation; **b** Posterior circulation; **c** Multiple circulations; **d** Border-zone territories. Single acute infarction. **e** Subcortical lesion with diameter ≤ 15 mm; **f** Subcortical lesion with diameter > 15 mm; **g** Corticosubcortical lesion; **h** Cortical lesion

All MR images collected from individual centers in digital format were read centrally by two readers (X.Z. and J.J.) blinded to the patients’ baseline and outcome information. Minor stroke patients with new infarction according to DWI were included in the final analysis. All minor stroke patients were classified as SAI or MAIs according to infarction numbers [5]. Uninterrupted lesions visible in contiguous territories were considered SAI, and more than one lesions topographically distinct (separated in space or discrete on contiguous slices) were defined as MAIs, according to previous DWI studies [5, 19]. According to previous studies [6, 7], SAI were also classified as lacunar infarction (subcortical lesion with diameter ≤ 15 mm) and non-lacunar infarction (subcortical lesion with diameter > 15 mm, cortical lesion and corticosubcortical lesion) and MAIs were classified as 1. Unilateral anterior circulation; 2. Posterior circulation; 3. Multiple circulations; 4. Border-zone territories (Fig. 1). Any disagreement was decided by a third reader (L.L.).

### Etiology classification

All patients were classified on the basis of The Trial of Org 10,172 in Acute Stroke Treatment (TOAST) classification [20] as previous study [5]. Patients with cardioembolism (CE), systemic disease were excluded in CHANCE, so there were no patients with stroke of CE or other determined pathogenesis subtype. Finally, we devided patients into three TOAST subtypes: large-artery atherosclerosis (LAA), small-artery occlusion (SAO) and stroke of undetermined pathogenesis. Subtype classifications were based on patients’ clinical features and the results of one or more diagnostic tests, including brain MR imaging, MRA and extracranial arteries (carotid ultrasound or computed tomograph angiography). All imaging data, clinical features and diagnostic tests results collected from individual centers were reviewed centrally by two study neurologists and gave the subtype classifications.

### Follow-up and outcomes

The original planed follow-up of the CHANCE trial was 90 days. However, we added a visit to follow patients for one year. All of follow-up visits were in person by a trained site coordinator. All reported outcomes were verified by a central adjudication committee which was blinded to the study-group assignments. The outcome was stroke recurrence (ischemic or hemorrhagic) during one-year follow-up [21]. We defined ischemic stroke as an acute focal infarction of the brain or retina with one of the followings: a new focal neurologic deficit lasting for ≥24 h, with clinical or imaging evidence of infarction and not ascribed to a nonischemic cause; sudden onset of a new focal neurologic deficit lasting for less than 24 h and not ascribed to a nonischemic cause, accompanied by new brain infarction on CT or MRI; or rapid worsening of an existing focal neurologic deficit lasting more than 24 h and not ascribed to a nonischemic cause, accompanied by new ischemic changes on CT or MRI of the brain and visibly distinct from the index ischemic event. We defined hemorrhagic stroke as acute extravasation of blood into the subarachnoid space or brain parenchyma with associated neurologic symptoms [17].

### Statistical analysis

Proportions were used for categorical variables, and medians with interquartile ranges were used for continuous variables. Univariate analyses were performed to compare the baseline characteristics among patients with different infarction patterns using one way analysis of variance or Kruskal-Wallis test for continuous variables and x^2^ test for categorical variables. Time to the event in each imaging group illustrated using Kaplan-Meier curve. We assessed the associations between infarction patterns and stroke recurrence of minor stroke using multivariable Cox regression models. Adjusted hazard ratios (HRs) with 95% confidence intervals (CIs) were reported. All the potential covariates listed in Table 1 were included in the model. All tests were two-sided, and a *P* value < 0.05 was considered to indicate statistical significance. All statistical analyses were performed with SAS 9.4 (SAS Institute Inc., Cary, NC).

**Table 1:** Baseline characteristics of single acute infarction (lacunar infarction and non-lacunar infarction) and multiple acute infarctions

Characteristics	Single acute infarction:lacunar infarction *n* = 381	Single acute infarction: non-lacunar infarction *n* = 172	Multiple acute infarctions *n* = 281	*P* value
Age,y, median (IQR)	62.6 (54.6–70.5)	61.0 (54.1–70.1)	64.8 (56.3–73.0)	0.008
Male, n (%)	261 (68.5)	105 (61.0)	193 (68.7)	0.17
Body mass index (kg/m^2^)	24.5 (22.7–26.2)	24.2 (22.0–26.6)	24.2 (22.0–26.2)	0.37
Medical history, n (%)				
Ischemic stroke	63 (16.5)	27 (15.7)	54 (19.2)	0.55
TIA	5 (1.3)	4 (2.3)	9 (3.2)	0.25
Myocardial infarction	6 (1.6)	4 (2.3)	9 (3.2)	0.38
Angina	9 (2.4)	0 (0.0)	9 (3.2)	0.07
Congestive heart failure	2 (0.5)	2 (1.2)	10 (3.6)	0.009
Hypertension	243 (63.8)	109 (63.4)	184 (65.5)	0.87
Diabetes mellitus	77 (20.2)	35 (20.3)	69 (24.6)	0.36
Hypercholesterolaemia	45 (11.8)	20 (11.6)	32 (11.4)	0.99
Current or previous smoking, n (%)	179 (47.0)	62 (36.0)	129 (45.9)	0.046
Time to randomization, n (%)				0.025
< 12 h	160 (42.0)	88 (51.2)	145 (51.6)	
≥ 12 h	221 (58.0)	84 (48.8)	136 (48.4)	
NIHSS on admission, median(IQR)	2.0 (1.0–3.0)	2.0 (2.0–3.0)	2.0 (1.0–3.0)	< 0.001
TOAST classification, n (%)				< 0.001
Large-artery atherosclerosis	127 (33.3)	82 (47.7)	183 (65.1)	
Small-artery occlusion	254 (66.7)	0 (0.0)	0 (0.0)	
Undetermined cause	0 (0.0)	90 (52.3)	98 (34.9)	
Group, n (%)				0.54
Aspirin only	195 (51.2)	89 (51.7)	133 (47.3)	
Clopidogrel+aspirin	186 (48.8)	83 (48.3)	148 (52.7)	
Medications, n (%)				
Antihypertensive	126 (52.5)	52 (48.2)	79 (42.9)	0.15
Antidiabetic	37 (48.1)	17 (48.6)	30 (43.5)	0.82
Lipid-lowering	25 (56.8)	14 (70.0)	20 (62.5)	0.60

*IQR* Interquartile range, *NIHSS* National Institutes of Health Stroke Scale, *TOAST* Trial of Org 10,172 in Acute Stroke Treatment

## Results

Among the 5170 patients, 1089 patients undergoing all the MR sequences as required at baseline were included in the CHANCE imaging subgroup. After excluding 255 patients without infarction, a total of 834 patients with minor stroke were included.

Table 1 shows the baseline characteristics of infarction patterns in lacunar infarction of SAI, non-lacunar infarction of SAI and MAIs. MAIs were more likely to be older, have a history of congestive heart failure and be shorter time to randomization of the trial treatment. Lacunar infarction of SAI were more likely to be smokers. Non-lacunar infarction of SAI were more likely to have higher NIHSS on admission. Additional file 1: Table S1 shows the baseline characteristics of different infarction patterns in SAI and MAIs respectively.

**Fig. 2:**
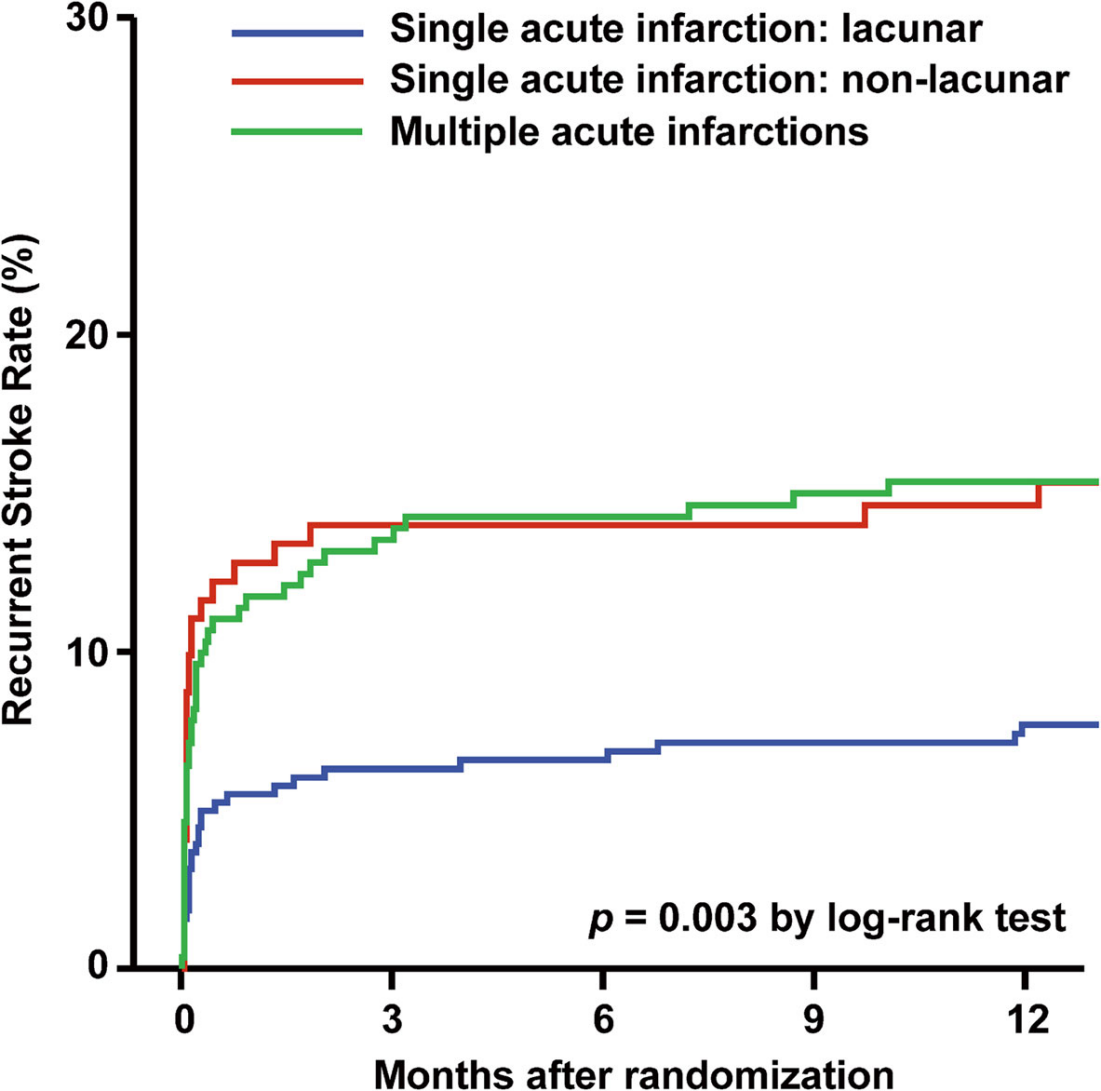
Stroke recurrence of single acute infarction (lacunar and non-lacunar infarction) and multiple acute infarction

Different infarction patterns in SAI (subcortical lesion with diameter ≤ 15 mm, subcortical lesion with diameter > 15 mm, cortical lesion and corticosubcortical lesion) had different risk of stroke recurrence (7.6%, 16.3%, 5.0% and 20.0%, respectively), however, different infarction patterns in MAIs (unilateral anterior circulation, posterior circulation, multiple circulations and border-zone territories) had no different risk of stroke recurrence (14.8%, 11.8%, 19.5% and 18.5%, respectively) (Additional file 1: Table S2). The risk of stroke recurrence was 7.6%, 15.1%, and 15.3% in patients with lacunar infarction of SAI, non-lacunar infarction of SAI and MAIs at 1 year follow-up, respectively (Table 2). Compared with MAIs, lacunar infarction of SAI had lower risk of stroke recurrence (HR 0.41, 95% CI 0.21–0.80, *P* = 0.009), but not in non-lacunar infarction of SAI (HR 1.01, 95% CI 0.60–1.69, *P* = 0.98) (Table 2). The Kaplan-Meier curves shows the recurrent stroke rate of SAI (lacunar and non-lacunar infarction) and MAIs, respectively (Fig. 2).

**Table 2:** Adjusted HR for stroke recurrence of different infarction patterns in single acute infarction and multiple acute infarctions at one-year follow-up

Infarction patterns	n	Stroke recurrence at one year		
		n (n% [95%CI])	Adjusted HR (95% CI)^a^	*P* value
Single and multiple acute infarctions	834	98 (11.8 [9.64–14.13])		
Multiple acute infarctions	281	43 (15.3 [11.30–20.05])	Ref	
Single acute infarction: lacunar	381	29 (7.6 [5.16–10.75])	0.41 (0.21–0.80)	0.009
Single acute infarction: non-lacunar	172	26 (15.1[10.12–21.36])	1.01 (0.60–1.69)	0.98

*HR* hazard ratio, *CI* confidence interval

^a^Adjusted for: age, sex, body mass index, history of ischemic stroke, TIA, myocardial infarction, angina, congestive heart failure, hypertension, diabetes mellitus, hypercholesterolaemia, smoking status, time to randomization, National Institutes of Health Stroke Scale on admission, Trial of Org 10,172 in Acute Stroke Treatment classification, group, antihypertensive medications, antidiabetic medications and lipid-lowering medications

## Discussion

In this subgroup analysis of CHANCE, we found that lacunar infarction of SAI had lower risk of stroke recurrence than MAIs, while non-lacunar infarction of SAI might have similar risk as MAIs within one-year follow-up.

TIA registry.org project showed MAIs had higher stroke recurrence than SAI in TIA or minor stroke [5]. However, former studies indicated there were more kinds of infarction patterns than that showed in TIA registry.org project [6, 7, 14]. Traditionally, SAI were classified according to the size and location of the infarction while MAIs were classified according to the blood supply of different brain areas [6, 7]. In our study, we found patients with different infarction patterns had different risk of stroke recurrence in SAI but the difference was not observed in MAIs. We inferred that significant difference of etiologies and pathogenesis among distinct infarction patterns led to the results.

Previous studies indicated that SAI with different patterns were usually related to different etiologies and pathogenesis. Lacunar infarction of SAI usually related to SAO with pathogenesis as ‘fibrinoid necrosis’ or ‘lipohyalinosis’ of small perforating arteries [22–24]. SAI with subcortical lesion with diameter > 15 mm usually related to large-artery atherosclerosis, cryptogenic and cardioembolic diseases [6, 7] with pathogenesis as obstruction of the origins of penetrating arteries by parent large intracranial artery intimal plaques or embolism [25–27]. Furthermore, SAI with corticosubcortical lesion or cortical lesion were usually related to LAA, CE and cryptogenic with pathogenesis of embolism [8, 9, 14]. In a word, lacunar infarction was different from non-lacunar infarction in aspect of etiologies and pathogenesis [28]. Traditionally, lacunar infarction usually had a favorable outcome among different TOAST classification [11, 12] and lacunar infarction had a favorable outcome when compared with non-lacunar infarction [29–31]. So the above findings could explain the different risk of stroke recurrence in different patterns of SAI for different etiologies and pathogenesis.

Previous studies indicated MAIs were usually related to LAA, CE and cryptogenic, according to the TOAST classification [6, 7]. There was evidence showed that the pathogenesis of MAIs was likely to be caused by the embolism from heart or major extracranial/intracranial vessels [6, 7, 32, 33]. Hemodynamic failure and microembolization were the pathogenesis of border-zone infarctions [34]. As embolism was the most common pathogenesis of MAIs, the above findings could explain the high and similar risk of stroke recurrence in patients with different infarction patterns of MAIs.

Recently, imaging parameters received more attention in order to predict recurrent stroke [3–5, 19] and might have better predictive value for stroke recurrence than clinical scores in patients with TIA or minor stroke [3, 35]. TIA registry.org project showed it was convenient and quick to stratify the risk of stroke recurrence by infarction numbers (NAI, SAI or MAIs) in clinical practice. However, our study indicated that non-lacunar infarction of SAI might have similar risk of stroke recurrence as MAIs, implying that non-lacunar infarction of SAI could be ignored if we simply stratified the risk of stroke recurrence by infarction numbers. So we should not only concern about the number of infarctions, but also the size and location of infarction in order to predict the risk of stroke recurrence in minor stroke. Improved infarction pattern classifications of TIA and minor stroke should be established in the future large cohort study.

Our study presented several limitations. First, since this imaging subgroup analysis included only a small part of patients of CHANCE, potential selection bias might have existed. Second, potential bias might have existed, as apparent diffusion coefficient was not included for evaluating infarction. Third, all patients in this imaging substudy were non-cardioembolic minor ischemic stroke which limited the generalizability of the findings to cardioembolic minor ischemic stroke.

## Conclusions

Lacunar infarction of SAI had lower risk of stroke recurrence than MAIs, while non-lacunar infarction of SAI might have similar risk as MAIs. Except for the number of infarctions, the size and location of the infarction should also be considered to stratify the risk of stroke recurrence in minor stroke.

## Additional file

Additional file 1: **Table S1.** Baseline characteristics of different infarction patterns in single acute infarction and multiple acute infarctions respectively. **Table S2.** Adjusted HR for stroke recurrence of different infarction patterns in single acute infarction and multiple acute infarctions at one-year follow-up. (DOCX 31 kb)

## Abbreviations

3D: 3-dimensional
CE: Cardio embolism
CHANCE: Clopidogrel in High-risk Patients with Acute Nondisabling Cerebrovascular Events
CI: Confidence interval
DWI: Diffusion-weighted imaging
HR: Hazard ratio
LAA: Large-artery atherosclerosis
MAIs: Multiple acute infarctions
MR: Magnetic resonance
MRA: Magnetic resonance angiography
NAI: No acute infarction
SAI: Single acute infarction
SAO: Small-artery occlusion
TOAST: The Trial of Org 10,172 in Acute Stroke Treatment
