# Supplementary material for: Does all single infarction have lower risk of stroke recurrence than multiple infarctions in minor stroke?
Source: BMC Neurol. 2019 Jan 8;19:7. doi: 10.1186/s12883-018-1215-0 (PMC6325885; doi:10.1186/s12883-018-1215-0)
Supplement: Supplementary file 1 — Table S1. Baseline characteristics of different infarction patterns in single acute infarction and multiple acute infarctions respectively. Table S2. Adjusted HR for stroke recurrence of different infarction patterns in single acute infarction and multiple acute infarctions at one-year follow-up. (DOCX 31 kb) [file 12883_2018_1215_MOESM1_ESM.docx]

**Table S1.** Baseline characteristics of different infarction patterns in single acute infarction and multiple acute infarctions respectively

| Characteristics | Single acute infarction | | | | *P*  value | Multiple acute infarctions | | | | *P*  value |
| --- | --- | --- | --- | --- | --- | --- | --- | --- | --- | --- |
|  | Subcortical lesion with diameter ≤ 15mm | Subcortical lesion with diameter > 15mm | Cortical lesion | Corticosubcortical lesion |  | Unilateral anterior circulation | Posterior circulation | Multiple circulations | Border-zone territories |  |
|  | n=381 | n=147 | n=20 | n=5 |  | n=162 | n=51 | n=41 | n=27 |  |
| Age, y, median (IQR) | 62.6 (54.6-70.5) | 59.8 (53.4-68.2) | 71.1 (63.6-77.3) | 62.3 (60.3-70.9) | 0.003 | 64.7 (55.8-72.5) | 68.0 (56.3-74.0) | 62.5 (56.8-71.0) | 68.2 (61.1-77.6) | 0.22 |
| Male, n (%) | 261 (68.5) | 88 (59.9) | 14 (70.0) | 3 (60.0) | 0.29 | 115 (71.0) | 35 (68.6) | 24 (58.5) | 19 (70.4) | 0.49 |
| Body mass index (kg/m^2^) | 24.5 (22.7-26.2) | 24.2 (22.0-26.5) | 23.4 (20.9-27.3) | 24.5 (24.0-27.0) | 0.51 | 24.2 (21.6-26.0) | 24.2 (22.9-27.1) | 24.2 (22.5-26.0) | 26.0 (22.5-27.2) | 0.54 |
| Medical history, n (%) |  |  |  |  |  |  |  |  |  |  |
| Ischemic stroke | 63 (16.5) | 16 (10.9) | 8 (40.0) | 3 (60.0) | <0.001 | 29 (17.9) | 8 (15.7) | 9 (22.0) | 8 (29.6) | 0.44 |
| TIA | 5 (1.3) | 3 (2.0) | 1 (5.0) | 0 (0.0) | 0.59 | 3 (1.9) | 4 (7.8) | 1 (2.4) | 1 (3.7) | 0.20 |
| Myocardial infarction | 6 (1.6) | 3 (2.0) | 1 (5.0) | 0 (0.0) | 0.71 | 5 (3.1) | 0 (0.0) | 2 (4.9) | 2 (7.4) | 0.31 |
| Angina | 9 (2.4) | 0 (0.0) | 0 (0.0) | 0 (0.0) | 0.25 | 6 (3.7) | 1 (2.0) | 0 (0.0) | 2 (7.4) | 0.35 |
| Congestive heart failure | 2 (0.5) | 2 (1.4) | 0 (0.0) | 0 (0.0) | 0.75 | 4 (2.5) | 4 (7.8) | 2 (4.9) | 0 (0.0) | 0.21 |
| Hypertension | 243 (63.8) | 92 (62.6) | 14 (70.0) | 3 (60.0) | 0.93 | 102 (63.0) | 37 (72.5) | 27 (65.9) | 18 (66.7) | 0.66 |
| Diabetes mellitus | 77 (20.2) | 31 (21.1) | 3 (15.0) | 1 (20.0) | 0.94 | 44 (27.2) | 9 (17.6) | 8 (19.5) | 8 (29.6) | 0.42 |
| Hypercholesterolaemia | 45 (11.8) | 18 (12.2) | 2 (10.0) | 0 (0.0) | 0.86 | 16 (9.9) | 7 (13.7) | 7 (17.1) | 2 (7.4) | 0.50 |
| Current or previous smoking, n (%) | 179 (47.0) | 54 (36.7) | 6 (30.0) | 2 (40.0) | 0.11 | 76 (46.9) | 23 (45.1) | 18 (43.9) | 12 (44.4) | 0.98 |
| Time to randomization, n (%) |  |  |  |  | 0.05 |  |  |  |  | 0.51 |
| <12 hours | 160 (42.0) | 71 (48.3) | 13 (65.0) | 4 (80.0) |  | 83 (51.2) | 23 (45.1) | 22 (53.7) | 17 (63.0) |  |
| ≥ 12hours | 221 (58.0) | 76 (51.7) | 7 (35.0) | 1 (20.0) |  | 79 (48.8) | 28 (54.9) | 19 (46.3) | 10 (37.0) |  |
| NIHSS on admission, median (IQR) | 2.0 (1.0-3.0) | 2.0 (2.0-3.0) | 2.0 (0.5-3.0) | 2.0 (2.0-2.0) | <0.001 | 2.0 (1.0-3.0) | 2.0 (0.0-2.0) | 2.0 (1.0-2.0) | 2.0 (1.0-3.0) | 0.17 |
| TOAST classification, n (%) |  |  |  |  | <0.001 |  |  |  |  | <0.001 |
| Large-artery atherosclerosis | 127 (33.3) | 67 (45.6) | 13 (65.0) | 2 (40.0) |  | 117 (72.2) | 28 (54.9) | 16 (39.0) | 22 (81.5) |  |
| Small-artery occlusion | 254 (66.7) | 0 (0.0) | 0 (0.0) | 0 (0.0) |  | 0 (0.0) | 0 (0.0) | 0 (0.0) | 0 (0.0) |  |
| Undetermined cause | 0 (0.0) | 80 (54.4) | 7 (35.0) | 3 (60.0) |  | 45 (27.8) | 23 (45.1) | 25 (61.0) | 5 (18.5) |  |
| Group, n (%) |  |  |  |  | 0.61 |  |  |  |  | 0.06 |
| Aspirin only | 195 (51.2) | 73 (49.7) | 13 (65.0) | 3 (60.0) |  | 80 (49.4) | 18 (35.3) | 25 (61.0) | 10 (37.0) |  |
| Clopidogrel+aspirin | 186 (48.8) | 74 (50.3) | 7 (35.0) | 2 (40.0) |  | 82 (50.6) | 33 (64.7) | 16 (39.0) | 17 (63.0) |  |
| Medications, n (%) |  |  |  |  |  |  |  |  |  |  |
| Antihypertensive | 126 (52.5) | 44 (48.4) | 6 (42.9) | 2 (66.7) | 0.77 | 40 (39.2) | 19 (51.4) | 13 (48.2) | 7 (38.9) | 0.56 |
| Antidiabetic | 37 (48.1) | 16 (51.6) | 0 (0.0) | 1 (100.0) | 0.26 | 17 (38.6) | 6 (66.7) | 3 (37.5) | 4 (50.0) | 0.45 |
| Lipid-lowering | 25 (56.8) | 13 (72.2) | 1 (50.0) | 0 (0.0) | 0.50 | 10 (62.5) | 4 (57.1) | 4 (57.1) | 2 (100.0) | 0.71 |

IQR: interquartile range; NIHSS: National Institutes of Health Stroke Scale; TOAST: Trial of Org 10172 in Acute Stroke Treatment.

**Table S2.** Adjusted HR for stroke recurrence of different infarction patterns in single acute infarction and multiple acute infarctions at one-year follow-up

| Infarction patterns | n | Stroke recurrence at one year | | |
| --- | --- | --- | --- | --- |
|  |  | n (n% [95%CI] ) | Adjusted HR (95% CI)^†^ | *P* value |
| Single acute infarction | 553 | 55 (9.9 [7.58-12.75] ) |  |  |
| Subcortical lesion with diameter ≤ 15mm | 381 | 29 (7.6 [5.16-10.75] ) | Ref |  |
| Subcortical lesion with diameter > 15mm | 147 | 24 (16.3 [10.75-23.31] ) | 3.33 (1.51-7.35) | 0.003 |
| Cortical lesion | 20 | 1 (5.0 [0.13-24.87] ) | 0.67 (0.08-5.38) | 0.71 |
| Corticosubcortical lesion | 5 | 1 (20.0 [0.51-71.64] ) | 3.42 (0.41-28.81) | 0.26 |
| Multiple acute infarctions | 281 | 43 (15.3 [11.30-20.05] ) |  |  |
| Unilateral anterior circulation | 162 | 24 (14.8 [9.73-21.24] ) | Ref |  |
| Posterior circulation | 51 | 6 (11.8 [4.44-23.87] ) | 1.09 (0.42-2.81) | 0.87 |
| Multiple circulations | 41 | 8 (19.5 [8.82-34.87] ) | 1.77 (0.72-4.37) | 0.22 |
| Border-zone territories | 27 | 5 (18.5 [6.30-38.08] ) | 1.37 (0.50-3.77) | 0.55 |

HR: hazard ratio; CI: confidence interval.

^†^Adjusted for: age, sex, body mass index, history of ischemic stroke, TIA, myocardial infarction, angina, congestive heart failure, hypertension, diabetes mellitus, hypercholesterolaemia, smoking status, time to randomization, National Institutes of Health Stroke Scale on admission, Trial of Org 10172 in Acute Stroke Treatment classification, group, antihypertensive medications, antidiabetic medications and lipid-lowering medications.
